# The Flavonoid Agathisflavone Attenuates Glia Activation After Mechanical Injury of Cortical Tissue and Negatively Regulates Both NRLP3 and IL-1β Expression

**DOI:** 10.3390/ijms26031275

**Published:** 2025-02-01

**Authors:** Verônica Moreira de Sousa, Áurea Maria Alves Nunes Almeida, Rafael Short Ferreira, Balbino Lino dos Santos, Victor Diogenes Amara da Silva, Jorge Mauricio David, Cleonice Creusa dos Santos, Silvia Lima Costa

**Affiliations:** 1 Laboratory of Neurochemistry and Cellular Biology, Institute of Health Sciences, Federal University of Bahia, Av. Reitor Miguel Calmon S/N, Salvador 40231-300, BA, Brazil; veumoreirasousa@gmail.com (V.M.d.S.); aurea.maria.almeida@gmail.com (Á.M.A.N.A.); rafa.short@gmail.com (R.S.F.); balbino.lino@univasf.edu.br (B.L.d.S.); vdsilva@ufba.br (V.D.A.d.S.); 2 College of Nursing, Federal University of Vale do São Francisco, Petrolina 56304-917, PB, Brazil; 3 Department of General and Inorganic Chemistry, Institute of Chemistry, Federal University of Bahia, Salvador 40170-110, BA, Brazil; jmdavid@ufba.br

**Keywords:** traumatic brain injury, astrogliosis, microglia, agathisflavone, neuroprotection, NRLP3, IL-1β

## Abstract

Traumatic brain injury (TBI) has a complex and multifactorial pathology and is a major cause of death and disability for humans. Immediately after TBI, astrocytes and microglia react with complex morphological and functional changes known as reactive gliosis to form a glial scar in the area immediately adjacent to the lesion, which is the major barrier to neuronal regeneration. The flavonoid agathisflavone (bis-apigenin), present in Poincianella pyramidalis leaves, has been shown to have neuroprotective, neurogenic, and anti-inflammatory effects, demonstrated in vitro models of glutamate-induced toxicity, neuroinflammation, and demyelination. In this study, we evaluated the effect and mechanisms of agathisflavone in neuronal integrity and in the modulation of gliosis in an ex vivo model of TBI. For this, microdissections from the encephalon of Wistar rats (P6-8) were prepared and subjected to mechanical injury (MI) and treated or not with daily agathisflavone (5 μM) for 3 days. Astrocyte reactivity was investigated by measuring mRNA and expression of GFAP protein in the lesioned area by immunofluorescence and Western blot. The proportion of microglia was determined by immunofluorescence for Iba-1; mRNA expression for inflammasome NRPL3 and interleukin-1 beta (IL-1β) was determined by RT-qPCR. It was observed that lesions in the cortical tissue induced astrocytes overexpressing GFAP in the typical glial scar formed and that agathisflavone modulated GFAP expression at the transcriptional and post-transcriptional levels, which was associated with a reduction of the glial scar. MI induced an increase in the proportion of microglia (Iba-1+), which was not observed in agathisflavone-treated cultures. Moreover, the flavonoid modulated negatively both the NRLP3 and IL-1β mRNA expression that was increased in the lesioned area of the tissue. These findings support the regulatory properties of agathisflavone in the control of the inflammatory response in glial cells, which can impact neuroprotection and should be considered for future studies for TB and other pathological conditions of the central nervous system.

## 1. Introduction

Traumatic brain injury (TBI) is a pathology with high morbidity, mortality, and permanent disability that is generally considered a silent epidemic with a major socioeconomic impact worldwide [1]. Due to the heterogeneity of pathogenesis, extent of injury, and diversity of cellular responses to damage, effective treatment strategies for TBI are lacking [2]. Several preclinical studies have focused on acute neuroprotection, broadly characterized by histological measures of cell death and lesion volume (Lerouet et al., 2021) [3]. Pharmacological treatment aims to prevent or reduce the acute and chronic effects of TBI. Tranexamic acid is a drug that can decrease the risk of death in patients with mild to moderate TBI if given early. Other treatments for TBI include anti-inflammatory agents, anticonvulsants, antidepressants, stimulants, and neuromodulators [4].

TBI is characterized as a complex and multifactorial injury that compromises the function of the central nervous system (CNS) and peripheral nervous system (PNS) caused via external force (such as traffic accidents, war, penetration of objects, explosions), classified as primary TBI and secondary injuries [5]. Primary injury events begin with mechanical trauma, axonal cuts, formation of bruises, and/or hemorrhages and can lead to disability or death in humans and animals [6]. Secondary lesions arise from the activation of cellular signaling pathways and molecular dynamics resulting in morphological and neurochemical changes, contributing to cellular/cytotoxic excitotoxicity, calcium overload, mitochondrial dysfunction, inflammation, and oxidative stress, as well as the mechanisms that influence the disruption of the blood–brain barrier (BBB)/vasogenic edema that characterize the pathology [7,8].

Astrocytes, the most abundant population of glial cells in the human brain, interact with several cells in the CNS and have a neuronal regulatory function, facilitating the progression of the degenerative process in days and months after the onset of the lesion by affecting other regions distinct from the primary lesion, such as the thalamus, hippocampus, medial septum, striatum, and amygdala [9]. The increase in astrocyte reactivity in response to injury is called astrogliosis. Reactive astrocytes show increased expression of intermediate filament proteins, mainly GFAP, as well as vimentin, and exhibit increased proliferation and secretion of growth factors, mainly glial-derived growth factor (GDNF), brain-derived growth factor (BDNF), and nerve growth factor (NGF), among others [10]. Astrocytes produce these neuroprotective peptides in the injured brain to clear cellular debris and orchestrate reparative and beneficial processes for neurological recovery after TBI [11,12]. On the other hand, when astrocytes are dysregulated, they produce cytotoxic mediators that hinder CNS repair by inducing neuronal dysfunction and cell death [13]. In addition to microglial activation, which leads to inflammasome activation, and astrocyte reactivity, immune cells from the peripheral system enter the blood–brain barrier (BBB) that has been damaged by mechanical trauma, potentiating neuroinflammation [14,15]. Progressive inflammation and cytokine release can induce a secondary brain injury process and consequently lead to a severe outcome, including death [16].

Flavonoids are polyphenestates derived from the secondary metabolism of plants and several studies have demonstrated their effects and potential for the treatment of neurodegenerative diseases and other pathologies of the CNS (for review see [17,18,19]). Agathisflavone (bis-apigenin) is a product of the oxidative coupling of two apigenins (4′,5,7-trihydroxyflavone) [20] and has aroused interest in scientific research due to its pharmacological potential [21]. This flavonoid has demonstrated positive effects, inducing neuronal differentiation and neurogenesis [22,23], and promising in vitro effects for pathological conditions of the CNS, including neuroprotective, antineuroinflammatory, and antitumor effects, as demonstrated in more recent studies [24,25]. In a study by Amorim et al. (2020) [26] using mixed cultures of the embryonic cortex subjected to mechanical injury as a trauma model, it was shown that treatment with agathisflavone reduced the expression of GFAP and thickening of astrocyte processes observed in the injured cultures and, consequently, reduced the glial scar, as well as inducing an increase in the number of neuron bodies and the growth of neurites that enter the area of the lesion, effects associated with the expression of NGF and BDNF growth factors. In order to better clarify neuroprotective and regenerative effects of the biflavonoid agathisflavone, in this study we investigated its effects and inflammatory mechanisms on astrocytes and microglia reactivity in brain slices subjected to mechanical injury.

## 2. Results

### 2.1. The Flavonoid Agathisflavone Modulates Astrocyte Reactivity in Brain Tissue Subjected to Mechanical Injury

**Figure 1:**
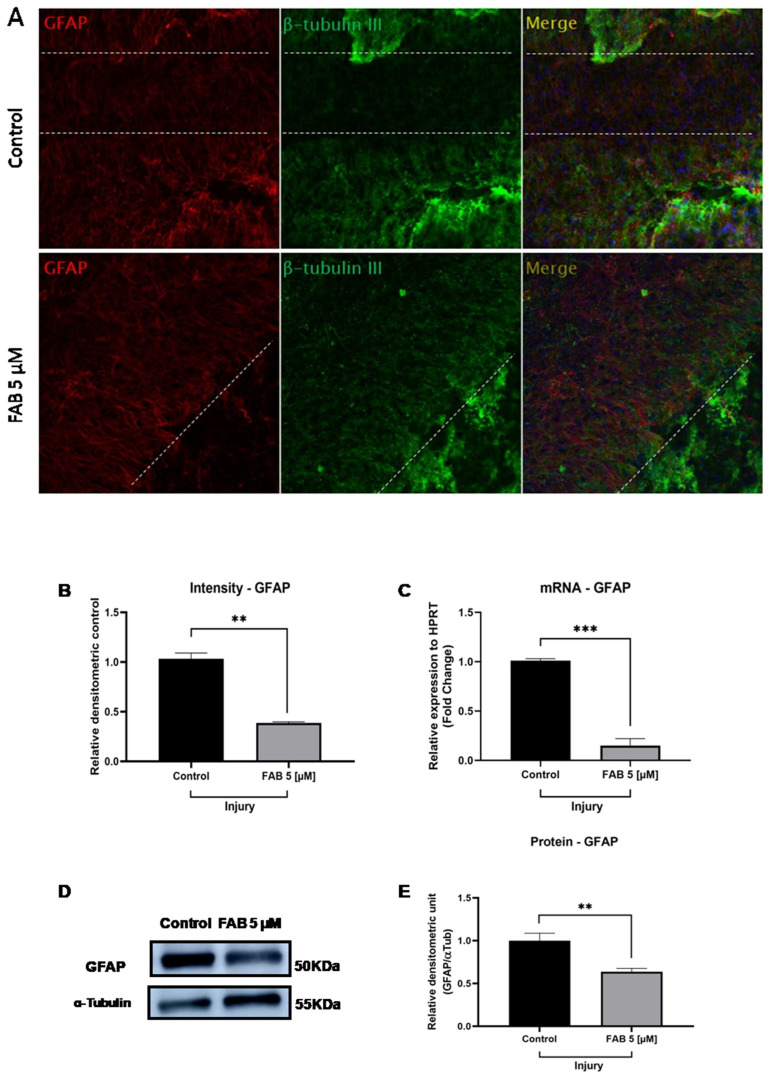
Analysis of astrocytic reactivity and neuron integrity in organotypic cultures of brain tissue from rats subjected to mechanical injury and treated or not with the flavonoid agathisflavone. The brain tissue slices were subjected to mechanical injury, treated with agathisflavone (FAB, 5 μM) or not (DMSO, 0.005%) at 24 h intervals for a total period of 72 h after the start of the experiment. (**A**) Representative photomicrographs of immunofluorescence staining for glial fibrillary acidic protein (GFAP) and β-tubulin IIII (β-Tub III) in the region subjected to mechanical injury in brain tissue slices in control conditions or treated with agathisflavone; scale bar = 200 µm; dotted lines indicate the edge of the injury. (**B**) The graph shows the quantification of Iba1+ cells expressed as a percentage of the total number of controls. (**C**) GFAP mRNA expression by RT-qPCR in brain tissue from rats subjected to mechanical injury. (**D**) Immunoreactive bands of GFAP and α-tubulin proteins in brain tissue from rats subjected to mechanical injury. (**E**) Relative expression of GFAP in brain tissue from rats subjected to mechanical injury. Values are expressed as the mean ± SEM (n = 3). Results were normalized against the intensity of the reference protein α-tubulin. Significance was determined using an unpaired *t*-test; ** *p* < 0.002; *** *p* < 0.0002.

The aim of this study was to characterize the profile of glial reactivity and neuronal integrity in brain tissue slices subjected to mechanical injury that were in control conditions (DMSO, 0.005%) or treated with agathisflavone (5 μM) every 24 h for a total period of 3 days after the beginning of the experiment, followed by the analysis of the expressions of the proteins GFAP, a component of the cytoskeleton and maker of astrocyte reactivity, and β-tubulin III, a component of the cytoskeleton of neurons. Immunofluorescence analysis revealed that the injury in brain tissue slices under control conditions induced the loss of neurites and astrogliosis, evidenced by the increase in cell volume and thickness of astrocytic processes, which was associated with the upregulation of GFAP expression, especially visible at the edges of the lesions (Figure 1A). In injured slices treated with agathisflavone, a modulation in the astrocytic response was observed, with the downregulation of GFAP expression in astrocytic processes that invaded the area of the injury from the edge, presenting with less thickness. There was also an increase in neuron bodies and the growth of neurites that migrated along the astrocytic processes that penetrated the injured area. A reduction in the fluorescence intensity of GFAP expression was observed in the group treated with agathisflavone compared to the control lesioned cultures (Figure 1B).

In order to confirm the immunofluorescence data, the expression of GFAP mRNA was analyzed by RT-qPCR, and GFAP protein expression was analyzed by Western blot. A significant reduction in GFAP mRNA expression was observed in cultures subjected to mechanical injury and treated with agathisflavone compared to untreated cultures (Figure 1C). Western blot analysis of GFAP expression revealed a significant reduction in GFAP levels in the lesion region of agathisflavone-treated cultures compared with the lesion region of untreated cultures (Figure 1D). These data indicate a downregulation of GFAP expression at the transcriptional and post-transcriptional levels in agathisflavone-treated brain slice cultures after mechanical injury.

### 2.2. The Flavonoid Agathisflavone Modulates Microglial Reactivity in Brain Tissue Subjected to Mechanical Injury

**Figure 2:**
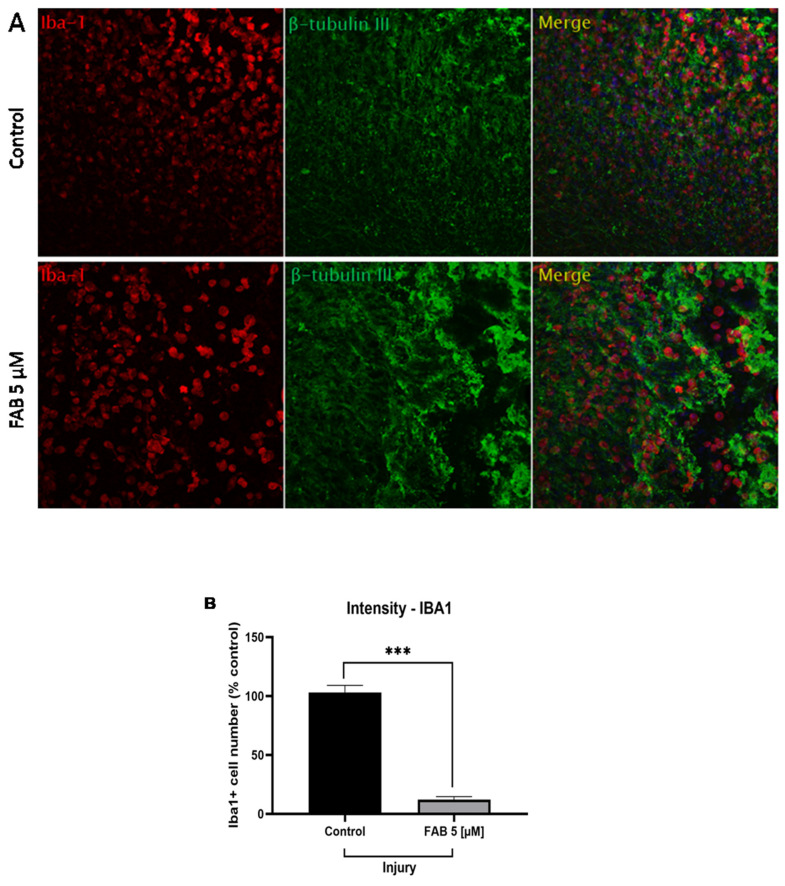
Analysis of microglial reactivity and neuron integrity in organotypic cultures of brain tissue from rats subjected to mechanical injury and treated or not with the flavonoid agathisflavone. The brain tissue slices were subjected to mechanical injury and treated with agathisflavone (FAB, 5 μM) or a control treatment (DMSO, 0.005%) at 24 h intervals for a total period of 72 h after the beginning of the experiment. (**A**) Representative photomicrographs of immunofluorescence staining for the microglia marker calcium-binding protein 1 (Iba-1) and marker of neuronal cytoskeleton components β-tubulin IIII (β-Tub III) in the region subjected to mechanical injury in brain tissue slices treated with agathisflavone at a concentration of 5 µM (FAB) or in control conditions (DMSO; 0.005%); scale bar = 200 µm. (**B**) The graph shows the quantification of Iba1+ cells expressed as a percentage of the total number of controls. Values are expressed as the mean ± SEM (n = 3). Results were normalized against the intensity of the reference protein α-tubulin. Significance was determined using an unpaired *t*-test; *** *p* < 0.002.

As demonstrated in Figure 2A, it was observed that injury in brain tissue slice cultures induced an increase in the number of microglia (Iba-1+ cells). However, treatment with agathisflavone (5 μM) induced a reduction in the proportion of microglia in the lesioned tissue (Figure 2A); this was also confirmed by measuring the fluorescence levels of this microglial protein in cultures subjected to injury but treated with the flavonoid agathisflavone (Figure 2B).

### 2.3. Agathisflavone Downregulates Interleukin 1β and NLRP3 Inflammasome mRNA Expression in Brain Tissue Subjected to Mechanical Injury

**Figure 3:**
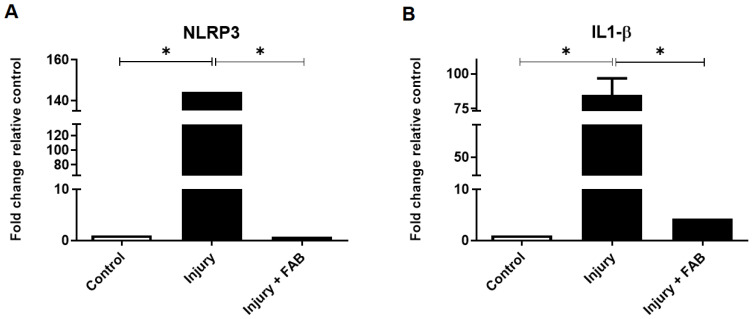
Effect of the flavonoid agathisflavone on the expression of NLRP3 inflammasome and interleukin-1β (IL-1β) in organotypic cultures of brain tissue from rats subjected to mechanical injury. The cultures were maintained under control conditions (DMSO 0.005%) or mechanically injured and or treated daily for 3 days with agathisflavone (FAB, 5 μM). The mRNA expression for NLRP3 (**A**) and IL-1β (**B**) was analyzed by RT-qPCR; C = control, region contralateral to the injury; values expressed as mean ± standard deviation; significant differences are expressed as (*) *p* ≤ 0.05 when comparing injury conditions and injury with treatment with the flavonoid (Injury + FAB). One-way ANOVA was followed by the Student–Newman post-test.

To investigate the molecular mechanisms that could be involved in the observed effects of agathisflavone in the modulation of astrogliosis and microgliosis associated with increased neuron counts in brain tissue slices subjected to mechanical injury, the expression of mRNA for cytokines and neurotrophins was examined using RT-qPCR (Figure 3). It was observed that the mechanical injury of brain slices upregulated the expression of IL-1β mRNA, as well as the mRNA of the NLRP3 inflammasome, compared to brain slices that were maintained in control conditions (DMSO 0.005%), and that treatment of injured brain slices with agathisflavone (5 μM) induced a significant reduction of these inflammatory markers.

## 3. Discussion

This study sought to characterize mechanisms of the effect of the flavonoid agathisflavone on the modulation of the astrocytic and microglial response in an ex vivo model of brain injury using brain tissue slices subjected to mechanical injury. Organotypic cultures of brain tissue are an important tool in the development of new therapies for TBI, considering, among other aspects, the preservation of brain structures, the possibility of investigating the behavior of cells involved in the response to physical damage, and the neuroinflammatory environment. The neuroprotective and immunomodulatory potential of the flavonoid agathisflavone has already been characterized in different models of damage associated with CNS pathologies in vitro, such as glutamate neurotransmitter excitotoxicity [23], inflammatory [24], and demyelinating damage [27], and an in vivo model of spinal cord injury (SCI) [25]. Studies in the literature show the neuroprotective activity of flavonoids and their ability to cross the blood–brain barrier, which have been demonstrated for the flavonoids hesperetin and naringenin (Youdim et al., 2003) [28], rutin and quercetin (Ferri et al., 2015) [29], and polyphenols in general (Figueira et al., 2017) [30]. Recently, we demonstrated in an in vivo model of spinal cord injury that agathisflavone alone at the doses tested protected injured spinal cord tissue and increased expression of neurotrophins, modulating the inflammatory response; this suggests that agathisflavone has the capacity in cross CNS barriers (Do Nascimento et al., 2022) [25].

Among the cells involved in the response to damage to brain tissue, including physical damage, astrocytes play a fundamental role considering their rapid response capacity, with morphological changes, molecular signaling, and the production of growth and inflammatory factors to contain the damage [31]. However, excessive activation can lead to inflammatory processes and worsen neuronal damage. Injuries of different degrees can stimulate different responses, where a severe injury is responsible for the formation of astrocytic scars [32,33]. It is already well established that astrocytic reactivity is characterized by an increase in volume and cellular processes associated with an increase in the expression of the intermediate filament protein GFAP, which makes up most of the glial scar surrounding the damage [34]. In this study, it was observed that agathisflavone was able to reduce GFAP expression both at the transcriptional and post-transcriptional levels in organotypic slices of cortex after traumatic stress. The study by Amorim et al. (2020) [26], developed in primary cultures dissociated from embryonic cortical tissue, also demonstrated that agathisflavone has the ability to reduce astrogliosis in vitro and increase the number of neuron processes in the lesion area in embryonic brain primary cultures, which was associated with an increase in neurotrophic factors such as NGF and GDNF.

Astrogliosis, due to morphological changes and molecular signaling, promotes BBB permeability and enhances the inflammatory process by interacting with microglia in inflammatory signaling [35]. Microglia are capable of assuming different activation phenotypes; they can be pro-inflammatory and anti-inflammatory or regulatory. One of the characteristics of microglia with a pro-inflammatory profile is the rapid proliferation and activation of the inflammasome pathway, with associated production of IL-1β [14,15]. As reviewed by Bortolotti et al. (2018) [36], several studies have shown that inflammasomes, mainly NLRP3, NLRP1, and AIM2, are involved in the generation of tissue damage and immune dysfunction after trauma. After recognition of trauma-induced DAMPs, inflammasomes participate in the development of an exaggerated systemic and specific inflammatory response in multiple ways, contributing to organ damage, such as the development of acute respiratory distress syndrome. Inflammasomes may also play a role in post-trauma immunosuppression mediated by dysregulated monocyte functions, compromising the body’s defense against infectious agents and increasing the risk of complications such as sepsis, shock, or multiple organ failure [37]. In this context, characterizing the involvement of inflammasomes as potential therapeutic targets is of great importance. In this study, in addition to the ability to regulate astrogliosis characterized by reduced GFAP expression and glial scarring, it was observed that the flavonoid modulated the proliferation of microglia in conditions of brain tissue trauma, effects associated with the modulation of the expression of the NLRP3 and IL-1β inflammasome. As already mentioned, studies have indicated the immunomodulatory capacity of the flavonoid agathisflavone against different insults and, more recently, the property of modulating the NRLP3 inflammasome in isolated microglia against LPS-induced damage was characterized, which had an impact on the protection of neurons [38]. Moreover, the capacity of agathisflavone in modulating inflammatory status and highly expressed miR146a and miR155 in β-amyloid/LPS-stimulated human microglia was demonstrated and associated with STAT3 signaling [39]. In addition to these studies, the neuroprotective effect of agathisflavone was demonstrated in an in vivo model of SCI [25] and, more recently, the property in modulating reactive astrogliosis and microgliosis was characterized in an in vivo model of trauma, effects associated with an increase in the neuroblast population at the subventricular zone [40]. Although our current study was limited to observing the effects of agathisflavone on the modulation of glial reactivity and expression of molecules associated with the inflammatory status and neuroprotection at a single time point, the findings, together with previous study, highlight the therapeutic potential of the compound for trauma conditions. Further studies over the longer term are required to determine whether agathisflavone promotes the generation of neuroblasts and neurons and the mechanisms involved in neurogenesis and the regulation of the glial cell response.

## 4. Materials and Methods

### 4.1. Organotypic Cultures

Organotypic cultures of brain slices from Wistar rats with a postnatal age of 6 to 8 days (P06 and P08) that had been subjected to mechanical injury were adopted as a model of brain trauma. The cultures were prepared according to the method of Stoppini et al. (1991) [41] with some modifications. For this purpose, the animals were decapitated and had their brains exposed, removed aseptically, and placed on a plate with dissection medium (HBSS), balanced salt solution composed of anhydrous CaCl_2_, KCl, NaCl, Glucose, KH_2_PO_4_, anhydrous Na_2_HPO_4_, MgCl_2.6_H2O, MgSO_4.6_H2O, and HEPES. Coronal brain slices with a thickness of 350 µm (two slices per animal) were obtained under sterile conditions with the aid of the McILwain Tissue Chopper apparatus, transferred to micropore membranes (Millicell–CM, Millipore, Bedford, MA, USA) accommodated in a six-well polystyrene plate, and cultured in minimum essential medium (MEM, Invitrogen) supplemented with HEPES, glucose, L-glutamine, phenol red, and 10% fetal equine serum (SFE) + HBSS. The plates were kept in an oven at 37 °C and with 5% CO_2_ for 30 min.

### 4.2. Flavonoid and Treatment

Then, the slices were treated with the biflavonoid agathisflavone (6,8″-Biapigenin; FAB), which was obtained from *Poincianella pyramidalis* (Tul.) L.P.Queiroz (syn. *Caesalpinia pyramidale* (Tul.) Gagnon & G.P. Lewis) leaves as described previously (Mendes et al., 2000) [20] and had a 99% purity. The flavonoid was diluted in dimethyl sulfoxide (DMSO, Sigma) in 100 mM stock solutions that were stored and protected from light at −4 °C. After 30 min of culture stabilization, a mechanical injury was made in each slice with the aid of a modified insulin needle through light pressure. Thereafter, the culture medium was changed to fresh medium containing an equivalent volume of dimethyl sulfoxide, the vehicle of flavonoid dilution, considered as the control (DMSO, 0.005%), or to fresh medium containing FAB (5 µM) and then incubated in the oven at 37 °C with 5% CO_2_. The medium was changed every 24 h with the same concentrations of DMSO or FAB for a period of 3 days (72 h). Experiments were performed in triplicate. The choice of agathisflavone concentration was based on previous studies conducted by our group that demonstrated that the flavonoid agathisflavone modulates the microglial neuroinflammatory response and enhances remyelination in mouse cerebellar slices [27]. On the third day, the supernatant was collected and the slices processed for experiments. The morphology of neurons and the state of activation of astrocytes and microglia (CNS immune effector cells) was evaluated by immunofluorescence; the expression of GFAP and GS astrocytic activation proteins was evaluated by Western blot; RT-qPCR was used to measure the mRNA expression of inflammatory markers.

### 4.3. Immunofluorescence Labeling

To evaluate the structures of effector cells (astrocytes and microglia) in organotypic cultures, immunofluorescence staining was performed using specific structural antibodies for neurons, such as β III-Tubulin (Biolegend, Sandiego, USA, 801202), astrocyte reactivation antibody, glial fibrillary acidic protein (GFAP) (DAKO, Z0334), and microglial ionized calcium-binding adaptor molecule-1 (Iba1) (Novus-NBP, 2-75397). Proteins were identified with secondary antibodies conjugated to fluorochromes (Alexa-Fluor 488 or Alexa-Fluor 596).

To perform immunofluorescence staining, after treatment, the cultures were washed three times with phosphate-buffered saline (PBS) and then incubated in paraformaldehyde (PFA 4%) for 1 h. After this time, the PFA was removed, and the cultures were washed three times with PBS. After washing, they were incubated for 12 h with 0.1% Triton X-100 in PBS (PBS-T) at 4 °C and then incubated for 3 h in blocking solution (in PBS-Triton X-100 0.1%, BSA 20%). Incubation was performed overnight with primary antibody solution (in PBS-T, 1% goat serum). After this time, the slices were washed 3 times in PBS-T and then incubated for 3 h in AlexaFluor-488 (green) secondary antibody solution (in PBS-T, 1% goat serum) for GFAP and Iba-1. The cell nuclei in all slices were stained with DAPI (Molecular Probes, Eugene, OH) for 10 min. After the incubation period, the slices were then washed three times and mounted with Fluoromount-G Mounting Medium (Invitrogen). Images were captured using a confocal spectral analysis scope (Leica TCS-SP8) with a 40× objective or a 20× objective, and a 63× oil reflection objective was used for more developed images. All experiments included cultures where the primary antibodies were not included, and nonspecific staining was not observed in such negative controls. 

### 4.4. Protein Assay and Western Blot

To analyze the expression of the GFAP astrocyte reactivity protein marker, after treatment, the slices were incubated with extraction buffer (4 mM urea, 2% SDS, 2 mM EGTA, 62.5 mM Tris-HCl pH 6.8, 0.5% Triton X-100) containing 1 µL/mL of a protease inhibitor cocktail (P8340). The protein concentration was determined using the BCA Kit (BIO-RAD) using the method of Lowry. A quantity of 2.5 µg of total proteins was subjected to polyacrylamide gel electrophoresis under 10% denaturing conditions (SDS-PAGE) and transferred to semi-dry PVDF membranes (Bio-Rad, Hercules, CA, USA).

The membranes were blocked with 5% nonfat dry milk (Molico) in tris-buffered saline containing 0.05% Tween-20 (TBS-T) for 1 h at room temperature under shaking. After blocking, the membranes were incubated with primary antibodies for the detection of GFAP (1:1000, Z0334 DAKO) or α-tubulin (1:1000, SC 23948, Santa Cruz) proteins. The membranes were then washed three times with TBS-T and incubated for 1 h at room temperature with a peroxidase-conjugated anti-rabbit or anti-mouse secondary antibody (1:10,000; G21234 Molecular Probes) diluted in 5% nonfat dry milk TBS-T. After three washes with TBS-T and one wash with TBS, the membranes were incubated with the chemiluminescent substrate solution (ECL Plus Bio Rad substrate kit) for 5 min. The immunoreactive bands were then analyzed using ImageQuant LAS 500 apparatus (GE Healthcare Life Sciences, Marlborough, MA, USA). After protein detection, semi-quantitative analyses were performed via densitometry using ImageJ software (Wayne Rasband, National Institutes of Health, Bethesda, MD, USA).

### 4.5. mRNA Expression Analysis by RT-qPCR

The total mRNA from organotypic cultures of brain slices exposed to different treatments was extracted using Trizol^®^ reagent (Thermo Fisher Scientific), following the protocol recommended by the manufacturer. The experiment was performed in biological triplicate. RNA quantification was performed using NanoDrop™ 2000 (ThermoFisher Scientific). The samples were stored in a freezer at −80 °C until use. For the cDNA reaction, 1.5 µg of RNA and the commercial High-Capacity cDNA Reverse Transcription Kit were used, as recommended by the manufacturer (ThermoFisher Scientific). The cDNA was stored at −20 °C until use. Subsequently, the quantitative real-time PCR (RT-qPCR) reaction was performed on ABI 7500 FAST equipment (Applied Biosystems) under the standard thermal cycling conditions for Taqman by the manufacturer. The mRNA expressions were evaluated in the treated samples and control conditions using commercial TaqMan^®^ probes: NLRP3 (Rn04244620_m1); IL1β (Rn00580432_m1); GFAP (Mm01253033_m1). The reference gene HPRT1 (Rn01527840_m1) (ThermoFisher Scientific) was used as a normalizer. The cDNA samples were diluted 1:100; 5 µL of TaqMan Universal Master Mix (Thermo Fisher Scientific) and 0.5 µL of specific Taqman^®^ probes were added for each monoplex reaction in a final volume of 10 µL. The expression of mRNA levels was calculated using the 2−ΔΔCT method, as described previously [38]) and analyzed using Graphpad Prism v 9.1.1 (2020).

### 4.6. Statistical Analysis of Results

Data were statistically analyzed using GraphPad Prism 8 software (GraphPad, San Diego, CA, USA) for Windows, expressed as mean and standard error of the mean or median and standard deviation. Statistical differences were considered significant for *p* ≤ 0.05. Results that presented a normal distribution were represented by the means and were subjected to normality tests, such as Shapiro–Wilk or D’Agostino–Pearson. Parametric statistical tests were used for comparisons between treatment and control groups. Results that were not normally distributed and were represented by medians were analyzed with nonparametric tests, such as the Mann–Whitney U test or Kruskal–Wallis.

## 5. Conclusions

In this study, the flavonoid agathisflavone, in an ex vivo model of TBI, showed an important effect in neuron preservation that was associated with the modulation of astrocyte and microglia reactivity, as well an anti-inflammatory effect via the regulation of microglial proliferation and expression of IL1β and the NRLP3 and inflammasome complex, important mediators of inflammation. These findings reiterate the regulatory properties of the inflammatory response of glial cells, which impacts neuroprotection against different insults and should be considered for future preclinical and clinical studies for CNS pathologies including TBI.

## Data Availability

Data are contained within the article.

## References

[1] Dias C., Rocha J., Pereira E., Cerejo A. (2014). Traumatic brain injury in Portugal: Trends in hospital admissions from 2000 to 2010. Acta Medica Port..

[2] Braun M., Vaibhav K., Saad N.M., Fatima S., Vender J.R., Baban B., Hoda M.N., Dhandapani K.M. (2017). White matter damage after traumatic brain injury: A role for damage associated molecular patterns. Biochimica et biophysica acta. Mol. Basis Dis..

[3] Lerouet D., Marchand-Leroux C., Besson V.C. (2021). Neuropharmacology in traumatic brain injury: From preclinical to clinical neuroprotection?. Fundam. Clin. Pharmacol..

[4] Tani J., Wen Y.T., Hu C.J., Sung J.Y. (2022). Current and Potential Pharmacologic Therapies for Traumatic Brain Injury. Pharmaceuticals.

[5] O’leary R.A., Nichol A.D. (2018). Pathophysiology of severe traumatic brain injury. J. Neurosurg. Sci..

[6] Stein D.M., Feather C.B., Napolitano L.M. (2017). Traumatic Brain Injury Advances. Crit. Care Clin..

[7] Simon D.W., McGeachy M.J., Bayır H., Clark R.S.B., Loane D.J., Kochanek P.M. (2017). The far-reaching scope of neuroinflammation after traumatic brain injury. Nat. Rev. Neurol..

[8] Jha R.M., Kochanek P.M., Simard J.M. (2019). Pathophysiology and treatment of cerebral edema in traumatic brain injury. Neuropharmacology.

[9] Yu G., Zhang Y., Ning B. (2021). Reactive Astrocytes in Central Nervous System Injury: Subgroup and Potential Therapy. Front. Cell. Neurosci..

[10] Pekny M., Wilhelmsson U., Pekna M. (2014). The dual role of astrocyte activation and reactive gliosis. Neurosci. Lett..

[11] Michinaga S., Koyama Y. (2019). Dual Roles of Astrocyte-Derived Factors in Regulation of Blood-Brain Barrier Function after Brain Damage. Int. J. Mol. Sci..

[12] Yuan M., Wu H. (2022). Astrocytes in the Traumatic Brain Injury: The Good and the Bad. Exp. Neurol..

[13] Michinaga S., Koyama Y. (2021). Pathophysiological Responses and Roles of Astrocytes in Traumatic Brain Injury. Int. J. Mol. Sci..

[14] Needham E.J., Helmy A., Zanier E.R., Jones J.L., Coles A.J., Menon D.K. (2019). The immunological response to traumatic brain injury. J. Neuroimmunol..

[15] Rehman S.U., Ikram M., Ullah N., Alam S.I., Park H.Y., Badshah H., Choe K., Kim M.O. (2019). Neurological Enhancement Effects of Melatonin against Brain Injury-Induced Oxidative Stress, Neuroinflammation, and Neurodegeneration via AMPK/CREB Signaling. Cells.

[16] Todd B.P., Chimenti M.S., Luo Z., Ferguson P.J., Bassuk A.G., Newell E.A. (2021). Traumatic brain injury results in unique microglial and astrocyte transcriptomes enriched for type I interferon response. J. Neuroinflamm..

[17] Costa S.L., Silva V.D., Dos Santos Souza C., Santos C.C., Paris I., Muñoz P., Segura-Aguilar J. (2016). Impact of Plant-Derived Flavonoids on Neurodegenerative Diseases. Neurotox. Res..

[18] Ayaz M., Sadiq A., Junaid M., Ullah F., Ovais M., Ullah I., Ahmed J., Shahid M. (2019). Flavonoids as Prospective Neuroprotectants and Their Therapeutic Propensity in Aging Associated Neurological Disorders. Front. Aging Neurosci..

[19] Bellavite P. (2023). Neuroprotective Potentials of Flavonoids: Experimental Studies and Mechanisms of Action. Antioxidants.

[20] Mendes C.C., Bahia M.V., David J.M., David J.P. (2000). Constituents of Caesalpinia pyramidalis. Fitoterapia.

[21] De Amorim V.C.M., Júnior M.S.O., Bastos E.M.S., Da Silva V.D.A., Costa S.L. (2018). Research on the Scientific Evolution of the Flavonoid Agathisflavone. J. Pharm. Pharm. Sci. A Publ. Can. Soc. Pharm. Sci..

[22] Paulsen B.S., Souza C.S., Chicaybam L., Bonamino M.H., Bahia M., Costa S.L., Borges H.L., Rehen S.K. (2011). Agathisflavone enhances retinoic acid-induced neurogenesis and its receptors α and β in pluripotent stem cells. Stem Cells Dev..

[23] Dos Santos Souza C., Grangeiro M.S., Lima Pereira E.P., Dos Santos C.C., da Silva A.B., Sampaio G.P., Ribeiro Figueiredo D.D., David J.M., David J.P., da Silva V.D.A. (2018). Agathisflavone, a flavonoid derived from Poincianella pyramidalis (Tul.), enhances neuronal population and protects against glutamate excitotoxicity. Neurotoxicology.

[24] de Almeida M.M.A., Souza C.D.S., Dourado N.S., da Silva A.B., Ferreira R.S., David J.M., David J.P., Costa M.F.D., da Silva V.D.A., Butt A.M. (2020). Phytoestrogen Agathisflavone Ameliorates Neuroinflammation-Induced by LPS and IL-1β and Protects Neurons in Cocultures of Glia/Neurons. Biomolecules.

[25] do Nascimento R.P., de Jesus L.B., Oliveira-Junior M.S., Almeida A.M., Moreira E.L.T., Paredes B.D., David J.M., Souza B.S.F., de Fátima D Costa M., Butt A.M. (2022). Agathisflavone as a Single Therapy or in Association With Mesenchymal Stem Cells Improves Tissue Repair in a Spinal Cord Injury Model in Rats. Front. Pharmacol..

[26] de Amorim V.C.M., Júnior M.S.O., da Silva A.B., David J.M., David J.P.L., de Fátima Dias Costa M., Butt A.M., da Silva V.D.A., Costa S.L. (2020). Agathisflavone modulates astrocytic responses and increases the population of neurons in an in vitro model of traumatic brain injury. Naunyn-Schmiedeberg’s Arch. Pharmacol..

[27] de Almeida M.M.A., Pieropan F., Footz T., David J.M., David J.P., da Silva V.D.A., Dos Santos Souza C., Voronova A., Butt A.M., Costa S.L. (2022). Agathisflavone Modifies Microglial Activation State and Myelination in Organotypic Cerebellar Slices Culture. J. Neuroimmune Pharmacol. Off. J. Soc. NeuroImmune Pharmacol..

[28] Youdim K.A., Dobbie M.S., Kuhnle G., Proteggente A.R., Abbott N.J., Rice-Evans C. (2003). Interaction between flavonoids and the blood-brain barrier: In vitro studies. J. Neurochem..

[29] Ferri P., Angelino D., Gennari L., Benedetti S., Ambrogini P., Del Grande P., Ninfali P. (2015). Enhancement of flavonoid ability to cross the blood-brain barrier of rats by co-administration with α-tocopherol. Food Funct..

[30] Figueira I., Garcia G., Pimpão R.C., Terrasso A.P., Costa I., Almeida A.F., Tavares L., Pais T.F., Pinto P., Ventura M.R. (2017). Polyphenols journey through blood-brain barrier towards neuronal protection. Sci. Rep..

[31] Escartin C., Galea E., Lakatos A., O’Callaghan J.P., Petzold G.C., Serrano-Pozo A., Steinhäuser C., Volterra A., Carmignoto G., Agarwal A. (2021). Reactive astrocyte nomenclature, definitions, and future directions. Nat. Neurosci..

[32] Yang T., Dai Y., Chen G., Cui S. (2020). Dissecting the Dual Role of the Glial Scar and Scar-Forming Astrocytes in Spinal Cord Injury. Front. Cell. Neurosci..

[33] Lima R., Monteiro A., Salgado A.J., Monteiro S., Silva N.A. (2022). Pathophysiology and Therapeutic Approaches for Spinal Cord Injury. Int. J. Mol. Sci..

[34] Benitz W.E., Dahl D., Williams K.W., Bignami A. (1976). The protein composition of glial and nerve fibers. FEBS Lett..

[35] Jassam Y.N., Izzy S., Whalen M., McGavern D.B., El Khoury J. (2017). Neuroimmunology of Traumatic Brain Injury: Time for a Paradigm Shift. Neuron.

[36] Bortolotti P., Faure E., Kipnis E. (2018). Inflammasomes in Tissue Damages and Immune Disorders After Trauma. Front. Immunol..

[37] 39- Latz E., Xiao T.S., Stutz A. (2013). Activation and regulation of the inflammasomes. Nat. Rev. Immunol..

[38] Dos Santos B.L., Dos Santos C.C., Soares J.R.P., da Silva K.C., de Oliveira J.V.R., Pereira G.S., de Araújo F.M., Costa M.F.D., David J.M., da Silva V.D.A. (2023). The Flavonoid Agathisflavone Directs Brain Microglia/Macrophages to a Neuroprotective Anti-Inflammatory and Antioxidant State via Regulation of NLRP3 Inflammasome. Pharmaceutics.

[39] Dos Santos B.L., Dos Santos C.C., da Silva K.C., Nonaka C.K.V., Souza B.S.F., David J.M., de Oliveira J.V.R., Costa M.F.D., Butt A.M., da Silva V.D.A. (2024). The Phytochemical Agathisflavone Modulates miR146a and miR155 in Activated Microglia Involving STAT3 Signaling. Int. J. Mol. Sci..

[40] Castro E Silva J.H., Pieropan F., Rivera A.D., Butt A.M., Costa S.L. (2024). Agathisflavone Modulates Reactive Gliosis After Trauma and Increases the Neuroblast Population at the Subventricular Zone. Nutrients.

[41] Stoppini L., Buchs P.A., Muller D. (1991). A simple method for organotypic cultures of nervous tissue. J. Neurosci. Methods.

